# ASO Author Reflections: Neoadjuvant Systemic Chemotherapy in Isolated Resectable Colorectal Peritoneal Metastases: Ready for Standard of Care?

**DOI:** 10.1245/s10434-022-11749-0

**Published:** 2022-04-12

**Authors:** Michael P. Flood, Robert Ramsay, Michael Michael, Alexander G. Heriot

**Affiliations:** 1 Department of Surgical Oncology, Peter MacCallum Cancer Centre, Melbourne, Australia; 2 Sir Peter MacCallum Department of Oncology, University of Melbourne, Melbourne, Australia; 3 Department of Medical Oncology, Peter MacCallum Cancer Centre, Melbourne, Australia

## Past

The diagnosis of colorectal peritoneal metastases (CRPM) was traditionally met with nihilism due to the lack of systemic chemotherapy efficacy and the perceived, futile efforts of surgical excision. With time, perceptions of CRPM biology began to shift from diffusely metastatic to locoregional, with the possibility of cure in selected patients through surgery. In 2003, the seminal RCT performed by Verwaal and colleagues^1^ placed cytoreductive surgery (CRS) with hyperthermic intraperitoneal chemotherapy (HIPEC) firmly on the map, though interestingly neoadjuvant systemic chemotherapy (NAC) had no role in the trial protocol. Single agent 5-fluorouracil (5-FU) was employed as adjuvant chemotherapy, which only 19 (35%) patients in the experimental arm completed successfully. Though the trial showed significant improvements in overall survival with surgery compared with chemotherapy alone, less refined patient selection and a now outdated systemic chemotherapy regimen are thought to be responsible for (what would be considered today) the low median survival in the experimental arm (22.3 months).

## Present

Advancements in medical oncology have led to the regular use of FOLFOX (5-FU and oxaliplatin), FOLFIRI (5-FU and irinotecan), or FOLFOXIRI (5-FU, oxaliplatin, and irinotecan) with adjunctive targeted therapy in metastatic colorectal cancer. Many of these regimens are often prescribed in neoadjuvant fashion for patients with resectable isolated CRPM in an effort to improve oncological outcomes by reducing disease burden and eradicating micrometastatic disease. This practice remains controversial given the lack of supporting clinical evidence.

In this systematic review and metaanalysis,^2^ we sought to assess the perioperative and oncological outcomes associated with the application of neoadjuvant systemic chemotherapy in patients with CRPM undergoing CRS + HIPEC. Firstly, patients who received NAC did not experience any increase in major perioperative morbidity or mortality compared with those who underwent upfront surgery. From an oncological perspective, there was no difference in disease-free survival between the groups, but on 5-year pooled analysis, NAC patients had improved overall survival (HR 1.31 95% CI 1.11–1.54, *p* < 0.001). Nevertheless, major limitations in the data, such as retrospective design and the lack of reporting on NAC patients who did not proceed to surgery, temper interpretation.

## Future

The challenging nature of prospective CRPM research and subsequent consensus/guidance has led to variance in institutional practices regarding perioperative (neoadjuvant and adjuvant) systemic chemotherapy. Certainly, from the PRODIGE 7 trial,^3^ where 83% of recruited patients received NAC, it would appear that clinical practice is heading towards NAC. The phase III trial of CAIRO6^4^ (which is actively recruiting) will provide the strongest evidence on perioperative chemotherapy: completion is expected in 2026.

As we continue to attempt to stratify and prognosticate patients with CRPM through biomarker discovery, the incentive to implement both personalized and standard lines of neoadjuvant systemic therapy into regular clinical practice will likely increase. While more robust evidence surrounding the application of NAC awaits publication, national/international guidance remains heterogeneous. Though we are not quite ready to call NAC “standard of care,” pending level 1 evidence may well confirm this.
